# Supplementary material for: Optical vortex-antivortex crystallization in free space
Source: Nat Commun. 2024 Jul 22;15:6178. doi: 10.1038/s41467-024-50458-y (PMC11263612; doi:10.1038/s41467-024-50458-y)
Supplement: Supplementary file 1 — Supplementary Information [file 41467_2024_50458_MOESM1_ESM.pdf]

Supplementary for "Optical vortex-antivortex crystallization in free space"

Haolin Lin[1,2,†], Yixuan Liao[1,2,†], Guohua Liu[1,2], Jianbin Ren[1,2], Zhen Li[1,2,3], Zhenqiang Chen[1,2,3*], Boris A. Malomed[4,5] and Shenhe Fu[1,2,3*]

[1] *Department of Optoelectronic Engineering, Jinan University, Guangzhou 510632, China*

[2] *Guangdong Provincial Key Laboratory of Optical Fiber Sensing and Communications, Guangzhou 510632, China*

[3] *Guangdong Provincial Engineering Research Center of Crystal and Laser Technology, Guangzhou 510632, China*

[4] *Department of Physical Electronics, Faculty of Engineering, Tel Aviv University, Tel Aviv, 69978, Israel*

[5] *Instituto de Alta Investigación, Universidad de Tarapacá, Casilla 7D, Arica, Chile*

A: Phase measurement of a 3×3 ionic-type lattice

In this section, we present an example for measuring the phase of the 3×3 square VAV lattice, using the method demonstrated in section of Methods. Specifically, we experimentally record the plane-wave interference pattern of the lattice at $z = 0$, with result presented in Fig. S1a. To improve measurement accuracy, we carefully adjust the carrier frequency k_c of the reference plane wave, such that we can separate the desired Fourier orders after Fourier transforming the interference pattern (Fig. S1b). Then we apply a square filter to extract the first-order Fourier component, as seen in Fig. S1c. The filter size should be properly adjusted, to optimize the signal-to-noise ratio of the obtained phase distribution. The inverse Fourier transform is performed on the exacted Fourier component that is shifted to the origin point in the frequency domain (Fig. S1e,f). Figure S1d depicts the recovered phase, revealing the polarities of individual vortices in the lattices. The measured phase is in accordance with the theoretical result, as shown in Fig. 2f in the main text, suggesting the validity of the presented phase measurement technique.

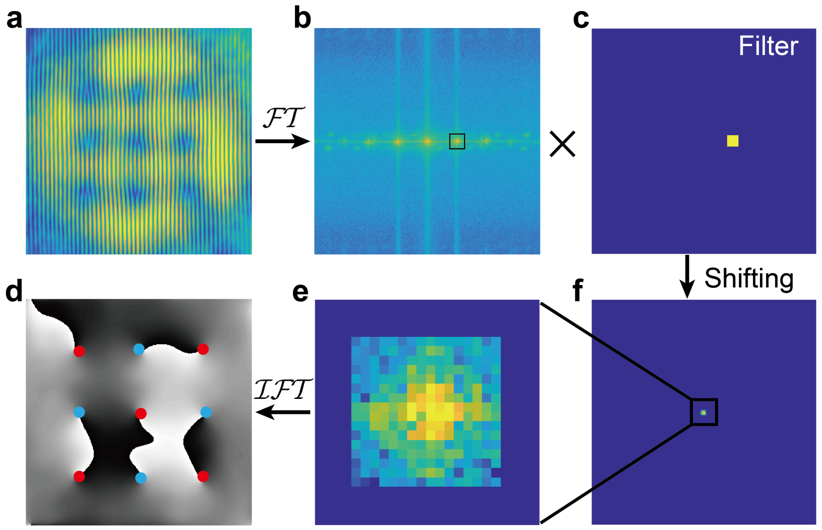

FIG. S1: **Illustration of phase measurement**. **a** The experimentally measured plane-wave interference pattern of the 3×3 VAV lattice. The experimental parameters are the same as those in Fig. 2 of the main text. **b** Fourier transform of the interference pattern. The black rectangle marks the desired Fourier component selected by a square filter in **c**. **d** The recovered phase distribution obtained by inverse Fourier transform of the filtered Fourier component (**e, f**). **e** The enlarged distribution of **f**. The red and blue circles in **d** represent vortex and antivortex pivots, respectively.

B: Phase-only holograms for generations of the ionic-type lattices

We construct the vortex-antivortex lattices which emulate the ionic arrangement of the solid-state crystal. To experimentally generate such lattices, it is required to fabricate the corresponding phase masks. To this end, we consider computer-generated holograms for encoding the amplitude and phase of the lattices by using the technique proposed by Bolduc (see Ref. [72] in the main text). Detailed procedures which enable producing the phase masks are summarized in the section of Materials and Methods of the main text. Figure S2a-c depict the produced phase masks for the 3×3, 5×5, and 7×7 lattices, which are loaded onto the spatial light modulator (SLM), see the experimental setup (Fig. 2a) in the main text. The vortex polarities for the pivots inside the lattice are visualized by the measured phases based on the method introduced in Materials and Methods. The measured phase distributions of these lattices are shown in Fig. S1d-f, respectively. Clearly, the expected alternating lattices are generated.

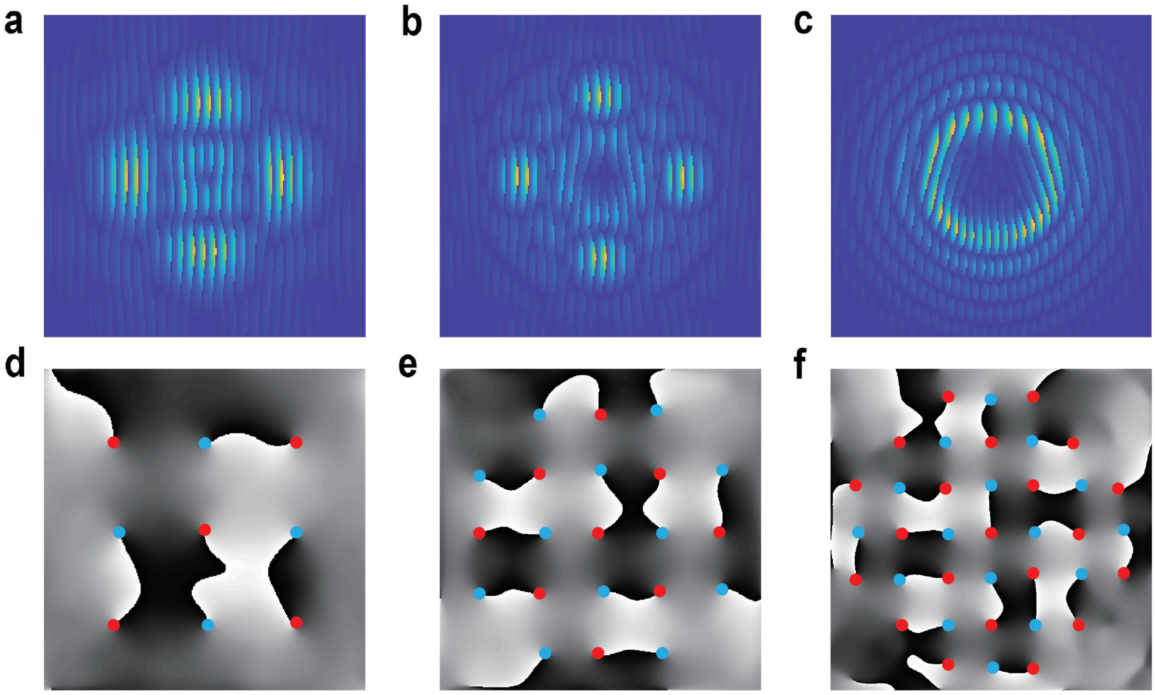

FIG. S2: **Phase-only holograms for VAV lattice generations**. **a-c** The phase-only masks fabricated for the creation of the stable vortex-antivortex lattices, which comprise 3×3 elements (**a**), 5×5 elements (**b**), and 7×7 elements (**c**), as displayed in the main text. **d-f** The experimentally measured phase distributions of the generated lattices. The lattice spacings for these three cases were measured to be $L = 320$ μm (**d**), $L = 310$ μm (**e**), and $L = 310$ μm (**f**), respectively.

C: Region of the ionic-type lattice spacing

The value of vortex-antivortex lattice spacing L is essential for the realization of stable lattices. To illustrate the issue, we consider theoretically two values of L for the nine-site lattices, in comparison to the case shown in Fig. 2c of the main text. First, we set the lattice spacing as $L = 0.8w$, where $w = 250$ μm denotes the waist of the Gaussian host field. We propagate the VAV lattice from $z = 0$ to $1.3z_R$, where $z_R = 310$ mm denotes the Rayleigh (diffraction) length. Figure S3a displays three-dimensional (3D) trajectories of the vortices and antivortices, see the red and blue lines, respectively. It shows that vortices and antivortices belonging to lattice edges attract each other and annihilate, initiating gradual disintegration of the lattice structure. Further simulations show that the vortices and antivortices annihilate at shorter distance for smaller values of L. The increase of the lattice spacing to $L = 2w$, while maintaining the same lattice structure, leads to trajectories of the pivots in the lattice shown in Fig. S3b, which clearly demonstrates the unstable propagation of the VAV lattice. In this case, the vortices and antivortices annihilate and then reappear, exhibiting repulsion in the course of the propagation. The instability of the propagation regime is explained by the attractions induced by the vortex-antivortex interactions. The most essential result is that the simulations reveal a range of values of the lattice spacing, $1.1w \leq L \leq 1.3w$, in which the interaction among the vortices and antivortices are balanced, resulting in the robust propagation of the VAV lattices, as demonstrated in the main text.

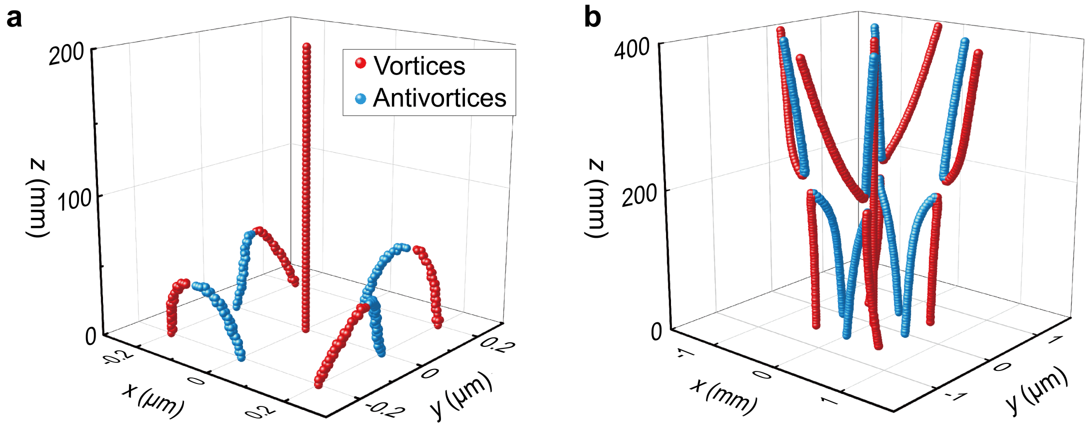

FIG. S3: Trajectories of the pivots in the 3×3 vortex-antivortex lattices for two different values of the lattice spacing: **a** $L = 0.8w$, and **b** $L = 2w$, where the width of the Gaussian envelope is $w = 250$ μm.

D: Propagation of a 3×3 lattice with identical vortices

We theoretically studied the propagation of a 3×3 square lattice composed of nine vortices with identical polarities, with the absence of antivortex. The lattice spacing is $L = 1.28w = 320$ μm, the same configuration as in Fig. 2c in the main text. The interference pattern of the initial lattice ($z = 0$) shown in Fig. S4a confirms that the pivots inside lattice are homopolar vortices. In this case, we observe rapid rotation of the lattice structure in the absence of the nonlocal orbit-orbit couplings, accompanied by gradually increasing separation of the vortex-antivortex spacing, as shown in Fig. S4b and S4c recorded at distances of $z = 200$ and 400 mm, respectively. Actually, the propagation dynamics of the homopolar lattice is determined solely by the uncoupling term F_0. Without contribution by the coupling term F_c, the vortices move along straight lines. The Pearson correlation coefficient [defined by Eq. (5) in the main text], as a function of z, is presented in Fig. S4d, confirming unstable propagation of the homopolar lattice. According to it, the lattice quickly disintegrates, as illustrated by the intensity pattern measured at distance of $z = 140$ mm (Fig. S4d), i.e. $z = 0.45z_R$, which is less than a half of the Rayleigh length.

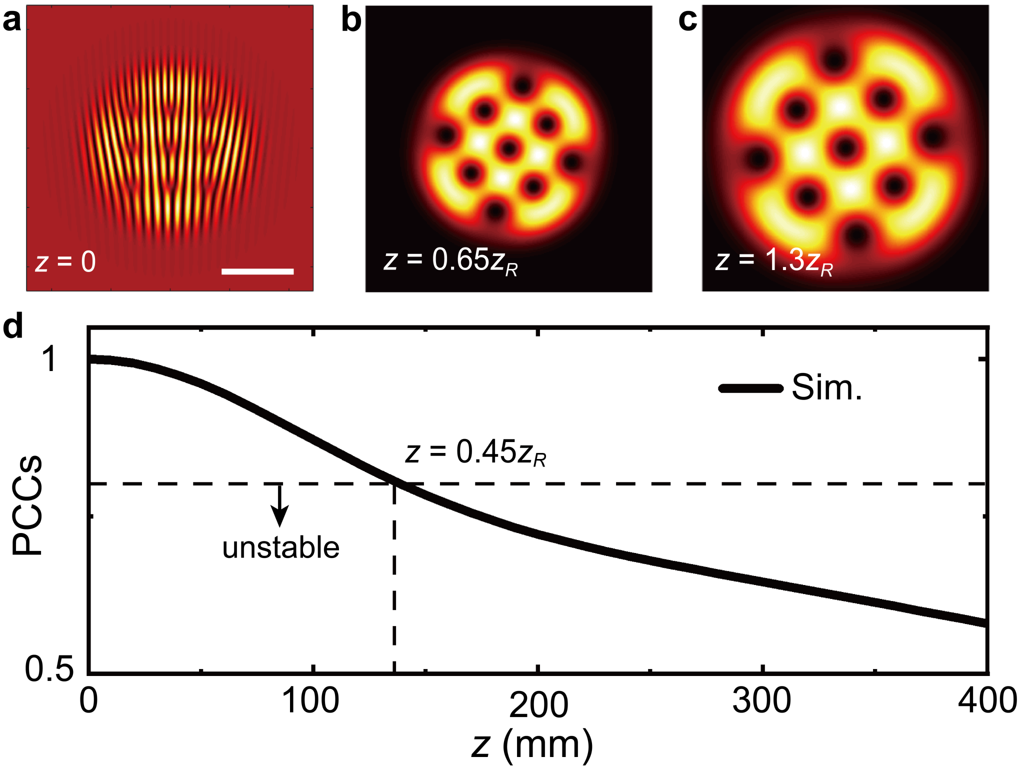

FIG. S4: **Unstable propagation of the 3×3 lattice built of homopolar vortices**. Parameters are the same as those in Fig. 2c of the main text. **a**, The interference pattern between the initial lattice ($z = 0$) and the plane wave, which shows the identical vortices nested in the Gaussian background. Panels **b** and **c** display intensity patterns at $z = 0.65z_R$ and $1.3z_R$, respectively. **d** The simulated Pearson correlation coefficient of the homopolar lattice as a function of the propagation distance. Panels **a-c** share the same scale, with scale bar being 0.60 mm.

E: Crystallization process of a 7×7 ionic-type lattice

We have included additional experimental results in Fig. S5, showing a crystallization process of a 7×7 vortex-antivortex lattice. Figure S5a shows the vortex-antivortex pattern at a distance of $z = -1.48z_R$, with a measured PCC value of 0.74 that indicates a nearly irregular lattice (Note that PCC below 0.8 indicates a disorder pattern); while Fig. S2b presents a measurement showing a generated regular vortex-antivortex lattice from the irregular one. The measured PCC value increases to 0.96. Figures S5c and S5d show the corresponding vortex and antivortex pivot distributions to Fig. S5a and S5b, respectively. The measurements directly demonstrate the crystallization process which transforms a disordered VAV lattice into a regular one. It is clear that vortices which are located at edge and far away from the center gradually move towards positions which the lattice site should have in the regular lattice, and then they stay in those positions in the course of long propagation.

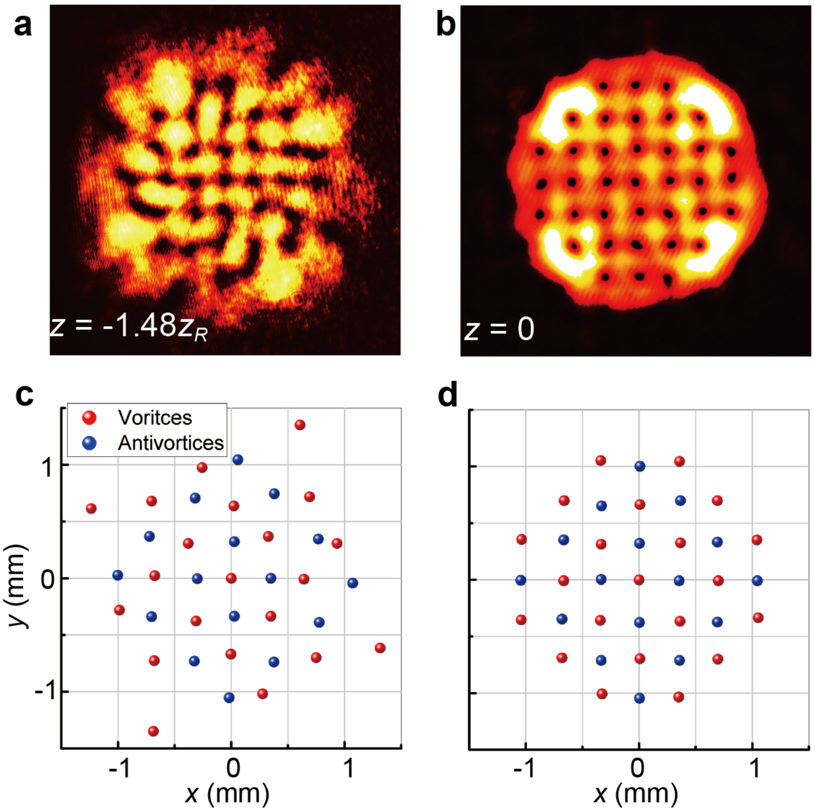

FIG. S5: **Additional measurements showing the vortex-antivortex crystallization of a 7×7 lattice**. **a** and **b** depict the measured intensity distributions of the light fields at $z = -1.48z_R$ and 0; while **c** and **d** illustrate their corresponding distributions of the vortex and antivortex pivots. The parameters used here are the same as those in Fig. 4 of the main text.
